# Food environment solutions for childhood obesity in Latin America and among Latinos living in the United States

**DOI:** 10.1111/obr.13237

**Published:** 2021-06-21

**Authors:** Ana Clara Duran, Melissa Mialon, Eric Crosbie, Melissa Lorena Jensen, Jennifer L. Harris, Carolina Batis, Camila Corvalán, Lindsey Smith Taillie

**Affiliations:** ^1^ Center for Food Studies and Research (NEPA) University of Campinas Campinas Brazil; ^2^ Center for Epidemiological Studies in Nutrition and Health University of São Paulo São Paulo Brazil; ^3^ Trinity Business School Trinity College Dublin Dublin Ireland; ^4^ School of Community and Health Sciences University of Nevada Reno Nevada USA; ^5^ Rudd Center for Food Policy and Obesity University of Connecticut Hartford Connecticut USA; ^6^ School of Nutrition, University of Costa Rica San José Costa Rica; ^7^ CONACYT, Health and Nutrition Research Center National Institute of Public Health Cuernavaca Mexico; ^8^ Instituto de Nutricion y Tecnologia de Alimentos University of Chile Santiago Chile; ^9^ Department of Nutrition, Gillings School of Global Public Health, and Carolina Population Center University of North Carolina at Chapel Hill Chapel Hill North Carolina USA

**Keywords:** childhood, food environment, obesity, policy

## Abstract

The food environment is a major contributor to unhealthy diets in children and, therefore, to the increasing rates of obesity. Acclaimed by scholars across the world, Latin American countries have been leaders in implementing policies that target different aspects of the food environment. Evidence on the nature and to what extent children are exposed and respond to unhealthy food environments in the region and among Latinos in the United States is, however, deficient. The objective of this review is to use the integrated International Network for Food and Obesity/noncommunicable diseases (NCDs) Research, Monitoring and Action Support (INFORMAS) framework to create healthy food environment to (i) compare the key elements of childhood obesity‐related food environments in Latin America and for Latinos living in the United States; (ii) describe the evidence on solutions to improve childhood obesity‐related food environments; and (iii) identify research priorities to inform solutions to fight childhood obesity in these populations. We found that an integrated body of evidence is needed to inform an optimal package of policies to improve food environments to which children in Latin America and Latino children in the United States are exposed and more efficiently translate policy solutions to help curb growing childhood obesity levels across borders.

## 1 INTRODUCTION

Children of Latino heritage in the United States and children living in Latin America have a high intake of sugar‐sweetened beverages (SSBs) and other ultra‐processed foods, key contributors to obesity,^1^ noncommunicable diseases (NCDs),^2^, ^3^ and mortality.^4^ A large body of evidence indicates that the food environment in which a person evolves contributes to unhealthy diets.^5^ Food environments have been defined as the “conditions that influence people's food and beverage choices and nutritional status” and include physical (e.g., availability, quality, marketing, and promotion), economic (cost), policy (rules), and sociocultural (e.g., social norms and preferences) dimensions.^5^ Promoting and protecting healthy food environments is particularly important for children, considering the links between the food environment and food preferences, behaviors, and appetite‐satiety signaling, which track into adolescence and adulthood.^6^

**FIGURE 1:**
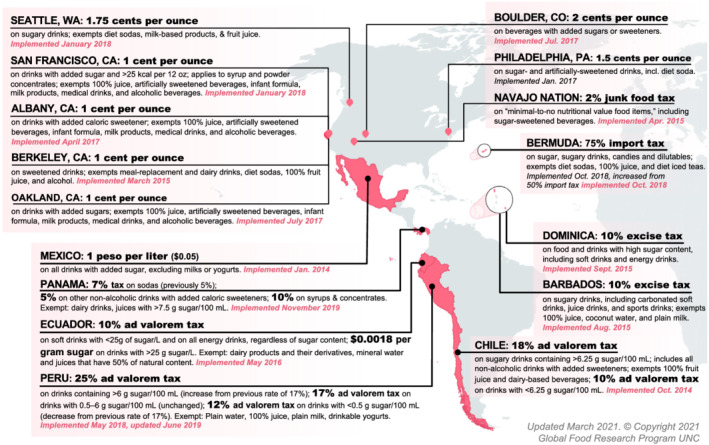
Taxation of sweetened beverages in the Americas, 2020

**FIGURE 2:**
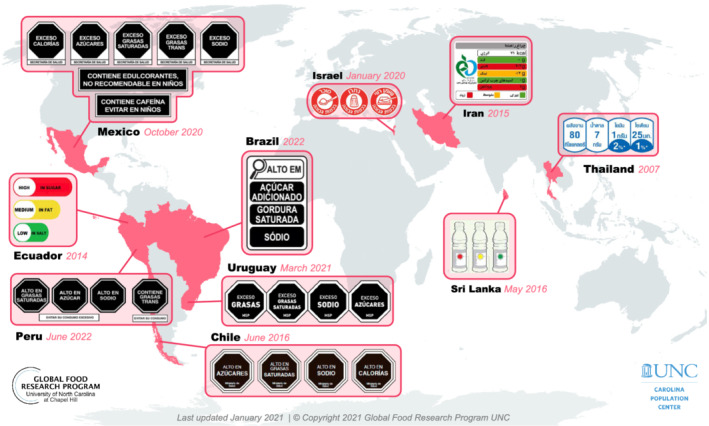
Countries with mandatory front‐of‐package nutritional labeling on packaged foods, 2020

Public policies and interventions that seek to protect and promote healthy food environments are important strategies to childhood obesity prevention and control.^7^, ^8^ Following the experience with tobacco control, policies such as increased taxation, marketing restrictions, and warning labels have recently been adopted to improve food environments as well.^9^, ^10^ These policies are usually aimed at reducing purchases of SSBs and other ultra‐processed foods.^11^, ^12^ In fact, countries in Latin America have been leaders in this realm. Mexico was the first country to implement taxes on both SSBs and selected ultra‐processed foods, with other countries following suit (Figure 1). Chile implemented the world's first mandatory front‐of‐package (FoP) warning label system,^13^ which has since been adopted across Latin America and elsewhere (Figure 2).

Yet, despite the extensive literature on the links between the food environment and diet, and the rapidly growing evidence on policies used to promote healthier food environments, less evidence is available for related issues concerning children, including Latin American children and children of Latino heritage living in other countries such as the United States.^14^ Preliminary evidence suggests similar challenges facing the two groups in the food environment; however, important differences are present. For instance, among Latinos living in the United States, acculturation and the length of time living in the United States can weaken ties to traditional diets from countries of origins and are associated with the consumption of ultra‐processed foods.^15^ Different governmental systems, norms and laws, and political viability to implement nationwide policies and interventions targeted at shaping food environments lead to greater or lower exposure to unhealthy food environments. However, because Latin American children and children of Latino heritage living in the United States share similar food cultures, health concerns, and, in many cases, food environment challenges, understanding whether and to what extent the food environments the children are exposed in both settings are similar can help shape more effective policies and population strategies.

**FIGURE 3:**
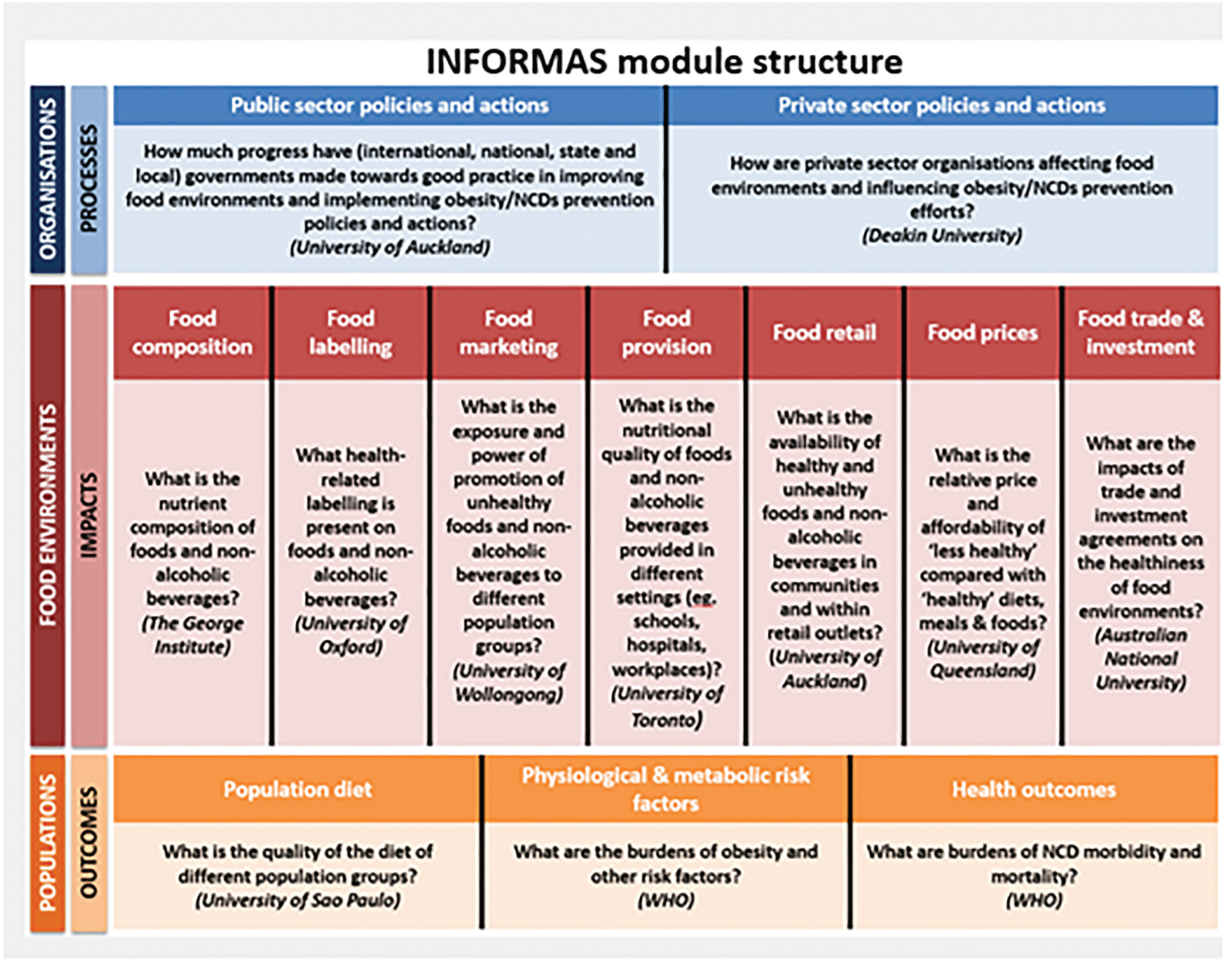
International Network for Food and Obesity/noncommunicable diseases (NCDs) Research, Monitoring and Action Support (INFORMAS)

The International Network for Food and Obesity/NCDs Research, Monitoring and Action Support (INFORMAS) provides a useful framework for identifying and monitoring key elements of the food environment and how they interact to produce effects on children's diet and health (Figure 3).^16^ This framework addresses key components of the food environment that are related to childhood obesity prevention, including food labeling, food promotion, food prices, food provisions in schools, private sector policies and actions, and food trade and investment. INFORMAS standardized methods used to assess different characteristics of the food environment have been conducted in 58 countries.^17^ These measures have contributed to capacity building, advocacy, and stakeholder engagement.^17^ INFORMAS standardized methods have also provided measures used in the evaluation of important food and nutrition policies related to food labeling, advertising,^9^ and pricing.^10^, ^18^

The objectives of the present review are to use the INFORMAS framework to compare and contrast the key elements of the food environment related to childhood obesity in Latin America and for Latinos living in the United States, describe the evidence on available solutions to improve childhood obesity‐related food environments, and identify research priorities to inform solutions that help reduce childhood obesity rates and that can be translated across borders.

## 2 FOOD LABELING

Food labeling refers to all elements present on a food or beverage product's package, including brand name or statement of identity, claims (e.g., health or nutrition related, structure or function, environmental, and social), nutrition information, and other marketing elements (e.g., child‐directed features such as licensed characters). Food labeling is important for healthy food environment because labels help inform and guide consumers in making healthier choices and, in some cases, incentivize the food industry to create healthier products.^19^

However, the information provided on food labels is complex and does not always help guide consumers towards the healthier choice. The nutrition facts panel (NFP) is typically the only mandatory nutritional information on a food label. However, not all countries require them. In countries where the NFP is required, the information may differ (e.g., whether added sugar, total sugar, or both is required). In addition, whether and how the NFP is used by consumers varies considerably. In the United States, about half of adults report using the NFP, although actual use may be lower. Latino adults are less likely to use the NFP.^20^, ^21^, ^22^ Similar inequalities have been found in Latin America.^23^, ^24^ In Brazil, adolescents who reported using the NFP had a lower consumption of ultra‐processed foods,^25^ but NFP use, and disparities in use, by children remains unclear. In both the United States and Latin America, consumers report the NFP to be confusing and that they prefer nutrition information on the front of the package.^26^, ^27^, ^28^

However, this is concerning, because other labeling elements on the front of the package, such as nutrition claims, can mislead consumers to think a product is healthier than it is.^29^, ^30^, ^31^ Nutrition claims are common in both the United States and Latin America, including on foods advertised to children.^32^, ^33^, ^34^ In both regions, studies have found that products with claims are more likely to have excess levels of nutrients of concern such as sugar or sodium than those that do not have nutrition claims.^33^, ^35^ Thus, reliance on nutrition claims may mislead consumers and contribute to purchases of SSBs and ultra‐processed foods.

### 2.1 Solutions and strategies

To address this problem of confusing nutrition labels, policies that require FoP labels on foods are increasingly common. Although FoP labeling models such as health stars or color‐coded traffic light labels have gained popularity in other parts of the globe, proposals in Latin America and the United States have focused primarily on placing FoP warnings on foods high in sugar, sodium, and saturated fat.^36^ Chile was the first country to implement a mandatory national FoP nutrient warning label policy in 2016,^37^ followed by Peru, Israel, Uruguay, and Mexico.^38^ Brazil has passed similar regulations, and a warning label law is under consideration by Colombia's congress. These warnings typically include text statements denoting high or excess levels of nutrients of concern, including added sugar, sodium, saturated fat, *trans* fat, and in some cases, energy, or low‐calorie sweeteners. The warnings also often, but not always, use shapes, text, or colors intended to signal a warning and to discourage consumption (i.e., a red stop sign or text that says, “avoid excess consumption”). The proposed mechanism is that the labels grab consumers' attention, elicit negative affect and thinking about the health harms of excess consumption, motivate behavioral intentions, and, ultimately, reduce purchases of SSBs and ultra‐processed foods.^39^ In the United States, no warning label legislation has been implemented, but nine jurisdictions have proposed health warnings on SSB containers, advertisements, or at the point of sale.^40^

A number of experimental studies in North America and Latin America related to nutrient warnings show that they help consumers identify unhealthy products,^41^, ^42^, ^43^ reduce intentions to purchase unhealthy foods,^41^, ^42^, ^44^, ^45^ and improve the healthfulness of purchases.^46^, ^47^, ^48^ In terms of real‐world evaluations, a recent study of SSB purchases after implementation of Chile's law found that SSB purchases declined by nearly 24% in the first 18 months, although Chile's law combines marketing and labeling policies.^9^ A qualitative study found that children can be a major mechanism for driving behavior change, with children learning at school what the labels mean, and asking their mothers not to purchase snacks or foods with the warning labels on them.^49^ Food reformulation after the law was enacted was observed, resulting in a 20% reduction in high‐sugar products and a 47% reduction in high‐sodium products.^50^

Concerns related to potential unintended consequences of reformulation in response to labeling policies remain, in particularly those related to low‐calorie sweeteners.^50^ These substances are found in at least 10% of the foods sold in Latin America and the United States.^51^, ^52^, ^53^ Despite international recommendations that discourage the consumption of low‐calorie sweeteners by children, these are found in foods advertised to Latin American children^54^ often without any information that could help caregivers identify their presence in foods and beverages.^55^ Consequently, 20% of Latino children and adolescents living in the United States report low‐calorie sweeteners consumption.^56^ Prior to the implementation of the Food Labeling and Advertising Law in Chile, low‐calorie sweeteners were found in the diet of up to 60% of children.^57^ Such consumption increased by 10% among preschoolers following the changes in nutritional labeling in that country.^58^ The long‐term consequences of the use of low‐calorie sweeteners at young ages include greater risk of developing NCDs as adults and long‐lasting preference for sweetened foods.^59^ In the short term, the consumption of low‐calorie sweeteners is associated with greater body mass index (BMI) and body fat.^60^

### 2.2 Research agenda

More detail about specific research questions can be found in Table A1. Broadly, a key area for additional research is understanding how FoP labeling efforts interact with other elements of food environment policies for children. For example, the Chilean experience suggests that the simultaneous restrictions on the sales and provision of foods high in critical nutrients inside schools were essential for parents to understand the FoP, but the mechanisms need further investigation. In addition, evidence on FoP impact is missing about US Latino parents and children, particularly those with low English literacy, which is important to understand if lessons learned from Latin America will apply in the US context.

## 3 FOOD PROMOTION

INFORMAS defines food promotion as “advertising, publicity and some sales promotions."^61^ The World Health Organization (WHO) cites “unequivocal evidence” that food and beverage marketing negatively impacts children's eating behaviors and body weight.^62^ Similarly, the Pan American Health Organization identifies ultra‐processed foods marketing as a significant contributor to children's risk for obesity and related diseases.^63^ Children's high daily exposure to food marketing, the poor nutritional quality of nearly all products marketed to children, and widespread use of unfair marketing techniques that take advantage of children's less developed cognitive abilities and other developmental vulnerabilities all raise concerns. The WHO calls for regulations to restrict marketing of foods and beverages to children (2–17 years) as a global health priority.^64^

In Latin America, the United States, and across the globe, research consistently documents high exposure to TV advertising for ultra‐processed foods, especially on children's programming and during peak viewing times.^65^, ^66^, ^67^, ^68^, ^69^, ^70^ Marketing tactics that disproportionately appeal to children, such as licensed cartoon characters, brand spokes‐characters, promotions, and fun/cool emotional appeals are common on TV and product packaging.^71^, ^72^, ^73^, ^74^ Research documenting other types of child‐directed food marketing is limited. However, TV advertising represents just one third of the $1.8 billion that US food companies spend on marketing directed at children (2–17 years).^75^ Furthermore, expenditures on digital marketing and other nonbroadcast marketing (including product placements, sponsorships, philanthropic promotions, and celebrity endorsements) have increased. In particular, digital marketing that targets children on mobile devices encourages viral sharing with peers, collects personal data, and blurs the distinction between advertising and entertainment in a way that is unfair and deceptive.^76^ In addition, companies utilize integrated marketing strategies designed to reach customers with a consistent message “everywhere.”^77^ In the United States, Latino‐targeted marketing also contributes to diet‐related health disparities affecting Latino communities.^78^ Food companies spent $800 million to disproportionately target advertising to Latino consumers on Spanish‐language TV.^79^ Furthermore, Spanish‐speaking youth visit food‐company websites^80^ and engage with more food brands on social media^81^ than white, non‐Hispanic youth.

### 3.1 Solutions and strategies

In 2010, the World Health Assembly recommended government regulation to reduce the impact of ultra‐processed foods marketing on children.^82^ In 2012, the WHO proposed an implementation framework with two policy approaches: comprehensive restrictions on all forms of ultra‐processed foods marketing to children or a stepwise approach restricting the most harmful types of marketing and/or products.^83^ More recently, the WHO recommended a comprehensive approach based on children's rights that incorporates ultra‐processed foods marketing with child appeals, including digital marketing, marketing in schools and at retailers, product packaging, product placements, and sponsorships. In fact, the protection of children's rights makes the need for regulations more urgent.

Despite WHO recommendations, most governments rely on industry self‐regulation to limit food marketing to children.^84^ The Children's Food and Beverage Advertising Initiative in the United States^85^ and the International Food & Beverage Alliance in Latin America are two examples.^86^ However, evaluations of industry self‐regulation consistently demonstrate little to no improvement following implementation primarily due to lax nutrition standards and limited coverage of different marketing strategies.^87^, ^88^ Furthermore, self‐regulatory policies only attempt to limit marketing to children under age 12 and do not address tactics that disproportionately appeal to younger children (e.g., licensed characters and promotions).^89^ Indeed, an evaluation of industry self‐regulation initiatives in the Americas rated them all as “low quality.”^90^

Government statutory policies are also limited and have primarily focused on regulating TV advertising or marketing in schools.^91^ For example, Mexico restricts ultra‐processed foods advertising during TV programming and the use of child‐directed marketing on product packaging, whereas Chile, Ecuador, Uruguay, and the United States prohibit in‐school marketing for nutrient‐poor products.^92^ Despite their limitations, statutory policies in Latin America are more likely to follow a child rights‐based approach, use nutrient profiling to identify ultra‐processed foods, cover a wider range of media platforms and settings beyond traditional TV, and address marketing techniques with child appeals compared with self‐regulation.^91^ The focus on children's rights, or ensuring that children are free from exploitation by food companies, is an important distinction underpinning the legal and political viability of food marketing policies in Latin America. In the United States, First Amendment protections on corporate speech make it more difficult to prioritize “children's rights.” For example, Brazil takes a broad child rights approach and prohibits “abusive publicity” intended to persuade children and adolescents to consume any product using strategies with child appeals. Enforcement of these restrictions is challenging. In fact, 80% of all food‐related ads shown on the three major Brazilian free‐to‐air TV channels included unhealthy foods and were largely from a handful number of national and transnational food companies and large supermarket chains.^93^

In 2015, Chile implemented the most comprehensive statutory policy to date prohibiting advertising of products with a high content of calories, saturated fat, sugar, and/or sodium on TV programming and websites with 20% or more children (under age 14) in the audience and child‐directed advertising on radio and in magazines.^91^ Chile also restricts promotional strategies and incentives with child appeals in product packaging, including licensed and brand characters, interactive games, and toys. Two years after the regulation was implemented, the prevalence of TV ads with foods high in critical nutrients decreased in TV programming primarily targeted to children, as well as for general audiences.^94^ Declines were also observed on the use of child‐directed strategies on cereal packages, accompanied by an increase in the consumption of breakfast cereals with less sugar.^73^ These studies will be instrumental to advance food marketing policies in other countries and to inform the development of effective policy solutions.

### 3.2 Research agenda

Further research is needed to assess the impact of comprehensive policies compared with policies that utilize a stepwise approach. In addition, evidence is lacking on the extent and impact of marketing aimed at children beyond TV advertising, including highly personalized forms of marketing (e.g., digital media) and integrated marketing strategies. A child rights‐based approach to food marketing policy requires research on the broader impact of child‐directed food marketing on children's rights (e.g., privacy and healthy development) and practices that take unfair advantage of their vulnerabilities. Evaluations of enacted policies should include pre‐ and post‐implementation measures to assess changes in children's exposure to marketing, purchases and/or consumption of regulated products, and on the overall diet. How and whether policies are delivered as expected and the food industry responses should also be included in evaluations. Previous regulations have led companies to reformulate products with questionable improvements (e.g., increasing the use of unregulated marketing practices). Finally, research must assess children's exposure to cross‐border marketing that originates in other countries without regulations.

## 4 FOOD PRICES

Food price and the relative price of healthy versus less healthy foods are important determinants of dietary intake, particularly among low‐income individuals.^95^ Evidence in the United States suggests that healthy foods and diets are more expensive than unhealthy ones and hence that cost is a barrier to eating healthy and a factor that contributes to obesity inequalities.^96^ However, methodological complexities make the evidence confusing and contradictory. For instance, when the unit of comparison for foods is energy ($/kcal), fresh produce seems much more expensive than energy‐dense snacks, whereas the opposite will be the case if the unit of comparison is the edible weight ($/g).^97^, ^98^ Moreover, analyses that evaluate the cost and quality of individuals' dietary intake cannot fully disentangle effects of food prices versus other factors that influence diet. In the United States, diet quality and cost are positively associated among non‐Latino populations, but not among Latinos.^99^ A possible explanation is that food price environments are different in Latino neighborhoods. A small study in Southern California found that prices of produce were lower in ethnic Mexican stores,^100^ whereas a study on supermarkets throughout the United States found no major difference between the healthy‐to‐unhealthy price ratio across neighborhoods with different proportions of Black/Latinos.^101^ Another explanation is that a more culturally acceptable healthy diet for Latinos is indeed not more expensive than an unhealthy diet even in the United States.

Evidence on the food price environment in Latin America is limited and complex. In Mexico, adults with more expensive diets had higher intakes of fruits, vegetables, and dairy, but also higher intakes of red meat, SSBs, and ultra‐processed foods, and a lower intake of beans.^102^ In Brazil, children with more expensive diets had higher intakes of essential micronutrients but an equal intake of fat and high‐sugar foods.^103^ Unhealthy food prices increased more than the price of healthier option in Mexico over the past few years (likely related to the 2014 Mexican tax),^104^ whereas in Brazil, the prices of ultra‐processed foods decreased.^105^ In Mexico, according to a modeling study healthy diets are less expensive; mainly because these have less meat and the cost associated with the increase in fruits, vegetables, grains, and legumes is equivalent to the savings resulting from the decrease in SSB and discretionary food purchase.^106^ In fact, fruits and vegetables are cheaper in Latin America than in high‐income countries.^107^

In sum, although increases in the cost of selected unprocessed and minimally processed foods have been observed in Latin America, it is likely that diets based on these foods are not necessarily more expensive than diets in which the share of ultra‐processed foods is high. Evidence suggests the same for Latinos living in the United States, but the question deserves further attention.

### 4.1 Solutions and strategies

Understanding whether the cost of healthier foods is a barrier for maintaining a healthy diet in different settings, particularly among racial/ethnic minorities, such as Latino children living in the United States is crucial. However, even if cost is not a barrier for healthy diets, fiscal policies are still valuable to counteract other factors promoting the intake of SSBs and other ultra‐processed foods such as convenience, marketing, and palatability. A meta‐analysis of prospective and intervention studies, mainly from the United States, found that a 10% decrease in the price of healthy foods (e.g., subsidy) increased their intake by 12%, whereas a 10% increase in price of unhealthy foods (e.g., tax) decreased their intake by 6%.^108^

To date, disincentives on unhealthy products, mainly SSBs, have been the most common fiscal strategy. SSB taxes have been implemented in Mexico, Chile, Barbados, Dominica, Ecuador, Peru, and many cities in the United States including Berkeley, Philadelphia, and Seattle (Figure 1). Available evaluations to date concur that taxes have accomplished the goal of decreasing intake or purchases of the taxed items^109^; although notably, few studies have examined taxes' effect on children and in the United States, no studies have examined specifically the impact on Latino populations. Furthermore, taxes could alleviate socioeconomic inequalities. In Mexico, individuals from lower socioeconomic status experienced a greater reduction in SSB purchases after the tax, suggesting that they can gain more health benefits. In Mexico, rural areas, which are poorer than urban areas, did not benefit from the tax as price increases were not passed to consumers.^110^, ^111^

A major gap remains with regards to fiscal policies that incentivize healthier purchases, as to date, evidence on price discounts or subsidies to healthy foods is limited to small‐scale interventions and randomized trials.^112^, ^113^

### 4.2 Research agenda

Further research on the associations between cost and healthfulness of foods in Latin America and among Latino populations in the United States is needed. Additional research needs include (i) evaluation of the impact of taxes on overall dietary intake, not only on taxed items or their direct substitutes; (ii) longer term evaluations to understand if and when consumers adjust to price changes and return to pretax purchasing levels; and (iii) whether these policies contribute to widening health inequalities, and what measures more effectively help mitigate unintended consequences.^113^ Although SSB taxes can help reduce income‐related health inequalities, this question is particularly important to address among US Latinos, because, to our knowledge, no studies have examined whether there is a differential impact of SSB or other taxes among Latino populations. Cross‐country research that compares taxes in the US to Latin American countries could be useful for addressing this question. Finally, pricing strategies have primarily focused on taxation of unhealthy foods. Large‐scale and empirical evidence on the effects of subsidies to healthy foods such as fruits and vegetables is lacking, as well as subsidies to commodities such as corn and soybean that disincentivize agricultural diversity and are used in ultra‐processed foods.^111^

## 5 FOOD PROVISION IN SCHOOLS

Typically, foods and beverages available at schools fall into three categories: those that are part of government‐administered school feeding programs (SFPs), competing foods usually provided by private vendors, and foods brought from home. Other aspects of the school food environment include the access to clean drinking water, the healthfulness of the food environment around schools, and the food marketing present within the schools.^114^

In the United States, the National School Lunch Program and the School Breakfast Program are available in public, charter, and nonprofit, private schools. These programs serve 30.4 million children daily, with lunches served free (66.6%) or at reduced price (6.7%) to students.^115^ Similarly, many countries in Latin America have a *Programa de Alimentación y Nutrición Escolar* or SFPs, which are typically administered by the country's Ministries of Education (Table 1). These programs vary widely across countries in their existence, coverage, resource allocation, administration, funding, and nutrition guidelines and reach more than 85 million students in the region.^116^ The Food and Agricultural Organization and the World Food Program have played important roles in shaping national SFPs by providing technical assistance based on the Brazilian experience. The Brazilian SFP dates back to the 1950s and has the largest reach in the world, serving over 40 million students daily.^117^ In 2009, Brazil was the first country in the world to pass a national law that made a farm‐to‐school component mandatory. This law requires that 30% of all foods procured for school meals must come from family farmers.^118^ In 2013, the Brazilian program's nutritional guidelines were strengthened by increasing the mandatory minimum number of servings of fruits and vegetables per week, prohibiting soda and a few other sugary drinks, and imposing maximum values for added sugar, fat, saturated fat, trans fat, and sodium in allowed foods. Recently, these regulations were updated with changes that aligned the procurement guidelines with Brazilian Dietary Guidelines.^119^ Passed in May 2020, the new regulation restricts the procurement of processed and ultra‐processed foods to 20% of federal funding.

**TABLE 1:** Programs and policies for food provision in schools by country

Country	School feeding program^a^			Mandatory regulation for competing foods and beverages sold inside schools^b^
	Name	Starting year	Coverage	
Bolivia	National Complementary School Meal Programme (PNACE)	2014	Universal coverage	
Brazil	Programa Nacional de Alimentación Escolar (PNAE)	1954	Universal coverage	Selected cities (2001–2013)
Chile	School Meals Programme (PAE)	1952	Targeted based on socioeconomic criteria	Yes (2016)
Colombia	School Meal Programme	1936	Targeted based on socioeconomic criteria	
Costa Rica	School Feeding Program for Children and Adolescents (PANEA)	1927	Universal coverage	Yes (2012)
Cuba	School Meals Programme	1970	Universal coverage	
Dominican Republic	School Meals Programme (PAE)	1997	Universal coverage	
Ecuador	School Meals Programme	1999	Universal coverage	Yes (2014)
El Salvador	School Meal and Health Programme (PASE)	1996	Universal coverage	
Guatemala	Support Programme: School Meals	1995	Universal coverage	
Haiti	National School Canteens Programme (PNCS)	1997	Geographic targeting	
Honduras	School Meals Programme	2000	Universal coverage	
Mexico	School Breakfasts	1929	Geographic and individual targeting	Yes (2011)
Nicaragua	Integral School Nutrition Programme (PINE)	1994	Universal coverage	
Panama	Complementary School Meals Programme	1995	Universal coverage	
Paraguay	Paraguay School Meals Programme (PAEP)	2014	Universal coverage	Yes (2013)
Peru	“Qali Warma” National School Meals Programme	2012	Progressive universal coverage	Yes (2015)
United States	National School Lunch Program (NSLP)	1946	Targeted based on socioeconomic criteria	

^a^ Information was obtained from WFP (https://www.wfp.org/publications/smart‐school‐meals‐nutrition‐sensitive‐national‐programmes‐latin‐america‐and‐caribbean), FAO (http://www.fao.org/3/a‐au438s.pdf), the Brazilian National School Feeding Program (*Programa Nacional de Alimentação Escolar*) (https://www.fnde.gov.br/programas/pnae), and the United States National School Lunch Program (https://www.fns.usda.gov/nslp/nslp‐fact‐sheet).

^b^ According to NOURISHING database.

In the United States, competing foods might include foods found in vending machines (e.g., chips and candy bars), “a la carte” items (e.g., pizza), and foods provided during in‐school celebrations and fundraisers. In Latin America, competing foods are usually those provided by private food vendors located inside schools that primarily sell ultra‐processed foods.^120^ A few Latin American countries have passed mandatory legislation aimed at restricting unhealthy competing foods and beverages sold inside schools.^121^ Mexico^122^ (2011), Costa Rica^123^ (2012), Uruguay^124^ (2013), Ecuador^125^ (2014), Perú^126^ (2015), and Chile^37^ (2016) have implemented such regulations, which differ in the policy instrument used (law, decree, or agreement), the nutrient cutoffs for defining healthy/allowed or unhealthy/prohibited foods, and mechanisms for compliance with the policy. Uruguay, Ecuador, Costa Rica, and Chile have regulations that prohibit the advertising of unhealthy foods inside schools^127^ (Table 1).

### 5.1 Solutions and strategies

In the United States, the Healthy Hunger‐Free Kids Act (HHFKA), passed in 2010, aimed to increase the availability of fruits and vegetables, whole grains, and fat‐free and low‐fat milk and to decrease the content of sodium, saturated fats, and trans fats in foods provided to children during lunch, breakfast, and snacks.^113^, ^128^ The overall nutritional quality of school meals was improved with the new HHFKA standards, although school lunch participation did not change.^129^

In Latin America, Brazil has taken a step further in improving school meals by restricting the procurement of processed and ultra‐processed foods served to school children in 2020, which deserves rigorous evaluations in forthcoming years.

Regulatory measures to ban sales of unhealthy competing foods inside schools have shown positive effects. A review focused on US policies found that laws and regulations influenced consumption or food availability in the expected direction, although effects on total dietary intake were less clear.^130^ A recent meta‐analysis quantified the impact of school food environment policies on dietary habits, adiposity, and metabolic risk in children and found that, although policies can improve targeted behaviors, the long‐term effects are unclear.^131^ In Chile, the availability of foods and beverages exceeding nutrient thresholds decreased after the policy was passed.^132^ In Mexico,^133^, ^134^ 2 years after the enactment of the guidelines, restricted foods were found in schools. Evidence from Costa Rica revealed the importance of selecting concrete and measurable objectives or goals and supporting stakeholders during the implementation process.^135^ For instance, food vendors continued to sell products that did not comply with the guidelines because they did not understand the regulation, to maintain profits, and as a result of poor enforcement.^135^

### 5.2 Research agenda

Rigorous evaluations of SFPs are needed, especially in Latin America where policies have been more comprehensive and included novel components like the requirement of foods from local farms (Brazil), restrictions on the procurement of ultra‐processed foods (Brazil), and banning marketing inside schools (Chile). Evaluations should monitor and measure the implementation and enforcement of such regulations and what has contributed to uptake and evaluate the impact of these policies on students' diets, educational outcomes, and health, and on the food system, including on the livelihood of family farmers.

## 6 FOOD RETAIL

The retail food environment, broadly defined as locations where people purchase food, including retail stores and restaurants, is probably the most widely studied component of the food environment in both the United States and Latin America. In both settings, different measures of the retail food environment have been associated with diet,^136^, ^137^ purchasing preferences,^138^ and obesity.^14^, ^138^, ^139^

Unequal access to healthy foods is also a reality in both settings, disproportionally affecting low‐income and racial/ethnic minority populations.^140^, ^141^ A wealth of studies considers proximity and exposure to large food retailers such as supermarkets close to home a proxy for access to healthy foods. However, in the United States, most individuals travel, on average, 6 km away from home to shop for food.^142^ Secondly, supermarkets are the main source of calories from ultra‐processed foods,^142^ and Latino families obtain the largest share of calories from grocery stores and supermarkets and the least from quick‐service and full‐service restaurants compared with other racial/ethnic groups.^143^ Similarly, the largest share of calories consumed by American children that come from selected ultra‐processed foods (e.g., SSBs, snacks, and grain‐based desserts) is purchased in grocery stores and supermarkets.^143^

In Latin America, the retail share of grocery stores and supermarkets varies by economic development and level of foreign direct investments.^144^ In Brazil, foods purchased at supermarkets account for 60% of the population's total energy intake and 60% of the calories that come from ultra‐processed foods.^145^ In other countries, such as Peru and Bolivia, where the participation of farmers' markets, produce markets, and other traditional retailers (butchers, tiendas, etc.) in the food supply is larger, this figure is likely to be lower.^144^ In fact, certain types of traditional retailers, like farmers' markets and produce markets, are recognized as important sources of unprocessed and minimally processed foods.^145^

Aside from the influence of the retail food environment on households with children, research suggests that children influence parents' purchasing behavior, both among Latinos in the United States^146^, ^147^ and in Latin America.^148^ Although parents report buying ultra‐processed foods to please their children in both settings,^148^ Latino children and parents mutually influence each other's choices at the point of purchase.^143^, ^144^ Additionally, Latino parents in the United States are more likely than white parents to bring their children with them when grocery shopping.^147^ A body of literature on different approaches to nudge consumers—including children—to make healthier choices inside food stores is growing sharply.^149^, ^150^, ^151^ For instance, changing placement of healthy and unhealthy foods, banning ultra‐processed foods from check out and placing them out of the reach of children, along with changes in promotional strategies of ultra‐processed foods are promising strategies but lack sufficient real‐world testing.^152^ More recently, researchers are employing innovative methods, such as the use of eye‐tracking technology,^150^, ^151^ consumer panels,^155^ and experiments using virtual^156^ and real‐life store labs to better understand children and caregivers' real‐world shopping behaviors and the uniqueness of stores that serve Latino populations.

### 6.1 Solutions and strategies

Studies that focused on the association between the retail food environment and childhood obesity specifically in Latino children in the United States and children living in Latin America are limited, lack robust designs, and have provided mixed results. One of the few studies employing a quasi‐experimental design using a sample of school‐aged children living in Arkansas, USA, found that the opening of a supermarket had a positive effect in low‐income children's BMI, but the closing of supermarkets did not alter children's weight.^157^ In the United States, a federal initiative to incentivize grocery stores and supermarkets to open in areas lacking access to nutritious, fresh food improved the availability of healthy foods but failed to show effects on diet,^158^, ^159^ except among Supplemental Nutrition Assistance Program (SNAP) participants.^160^ This finding depicts a potential synergistic effect of supply‐ and demand‐side interventions in improving diet that deserves to be further studied. Similarly, stocking improvements that followed the recent revisions of the Special Supplemental Nutrition Program for Women, Infants, and Children (WIC) have been observed in many US cities,^161^ they did not vary by neighborhood racial/ethnic composition^162^ and seemed to improve dietary intake.^161^

Less is known about the impact of retailer‐based policies in Latin America. Mexico's recent ban on the sales of ultra‐processed foods and SSBs to children under 18, recently implemented in the state of Oaxaca, is promising. Although the impact of this ban on children's purchases and intake has yet to be evaluated, evidence from similar tobacco control measures^163^ and bans on sales of electronic cigarettes to children and adolescents^164^, ^165^ suggests that such a ban can reduce ultra‐processed food purchases.

Finally, the role of produce or farmers markets in Latin America in the provision of unprocessed and minimally processed foods deserves to be explored, which may have been enhanced during the restricted phases of the COVID‐19 pandemic.

### 6.2 Research agenda

More research is needed to understand (i) the impact of food retailers, and different food retailer types, on the healthfulness of purchases of Latin American children and parents, and how this differs across countries, with robust statistical methods to deal with concerns relating to endogeneity and selectivity of retailer's decisions to locate in particular places; and (ii) interventions to improve the healthfulness of purchases within retailers (e.g., banning ultra‐processed foods in checkout), as well as whether the impact of these interventions and policies differs for Latino children and parents in the United States and in Latin America.

## 7 POLITICAL PRACTICES OF THE FOOD INDUSTRY

According to INFORMAS, the food industry includes those actors involved in producing, packaging, distributing, and marketing foods and beverages, as well as entertainment companies, the media, and other third parties working with them.^166^ There are numerous ways in which the food industry might influence food environments and policy solutions to improve their healthiness, including through (i) building strategic alliances with communities, the media, and other third parties outside and inside the industry; (ii) influencing science and information; (iii) directly influencing policy; (iv) using legal actions to prevent the adoption of mandatory regulations or to intimidate and destabilize opponents; and (iv) using argument‐based, discursive strategies that would favor food industry actors and their preferred solutions, sometimes at the detriment of public health.^167^, ^168^, ^169^

Children, including those in Latin America and Latino children in the United States, are directly targeted by corporations in the food industry, most notably through community initiatives.^170^ These initiatives often purport to support obesity prevention and put a particular emphasis on personal responsibility and physical inactivity,^168^, ^170^, ^171^ shifting the blame onto individuals for their ill‐health, while usually avoiding the question of the healthiness (or not) of food products. In Colombia, food companies engage in SFPs and community feeding programs by distributing unhealthy packaged foods to children.^172^ These corporate initiatives are sometimes carried out in partnership with local and national authorities^172^ and may certainly help food industry actors portray themselves through a positive light in communities and among policymakers.^168^ This is particularly important for the industry when it is at risk of being regulated. For instance, in Colombia, when a new FoP was discussed in Congress, an “Alliance for Child Nutrition” was launched by the food industry in the presence of the first lady of Colombia.^174^ The Alliance was then supported by the Attorney General, the Ministry of Health, the Presidential Council for Children and Adolescents, and the National Association of Neonatology.^174^

Food companies also influence the science on childhood obesity in Latin America, with Coca‐Cola, for example, funding large projects and local researchers on that topic,^175^ despite the clear vested interests the company has in the area. In Colombia, food companies' employees provide training to health professionals working with children as part of a national program.^172^ In Chile, food companies regularly sponsor pediatrics and nutrition annual congresses.^176^

These political practices to influence public policy, research, and practice in Latin America^172^, ^174^, ^177^ are consistent with those used by food industry actors around the world,^178^, ^179^, ^180^, ^181^, ^182^ including the United States.^168^ Today, in isolation and when combined, these practices are a major obstacle in the protection and promotion of healthy diets and in the fight against childhood obesity.^168^, ^169^, ^170^

### 7.1 Solutions and strategies

Raising public awareness about the food industry political practices is an important first step. Better protection of whistleblowers and public health advocates in their work in trying to protect and promote healthy diets and foods environments is also warranted. Public health professionals, government officials, and teachers should assess the risks when collaborating with food industry actors, particularly with initiatives carried out in communities and targeted at vulnerable populations, like children. Finally, food industry actors and their allies with a vested financial interest should not participate in the decision‐making stage of public health policy, as suggested by the WHO.^181^, ^182^

### 7.2 Research agenda

There are four main areas of research on the political practices of the food industry that could be explored in order to protect child health and prevent and control childhood obesity:^173^, ^181^ (i) continued identification and monitoring of the political practices of corporations; (ii) more research on similarities and differences in practices used by food industry actors when targeting Latino children in the United States compared with children in Latin America; (iii) benchmarking of governments, who have a duty to protect and promote population and child health and rights, and other actors like those in academia and professional associations, for efforts in trying to address and manage the political influence of the food industry; and (iv) better understanding of upstream, global drivers of ill‐health, such as neoliberal policies and capitalism, and their role in facilitating the use of political practices by food industry actors.

## 8 FOOD TRADE AND INVESTMENT

Trade liberalization, defined as the removal of trade barriers (e.g., tariffs and capital controls), has altered food systems globally by (i) opening domestic markets to foreign direct investment and international food trade, (ii) allowing food and beverage companies to enter markets and expand globally, and (iii) permitting increased global food and beverage advertising.^185^ This has especially affected low‐ and middle‐income countries, which have increasingly relied on food imports as their main source of food and diet.^186^

As trade agreements have evolved, they have become less about actual trade (e.g., importing/exporting restrictions) and increasingly more about investment.^187^ Trade agreements increasingly contain investment provisions that provide added protections for investors (e.g., corporations) such as intellectual property (e.g., trademarks, patents, and copyright) protections.^188^ Transnational corporations, including food and beverage companies, have lobbied and helped draft the terms of these agreements, altering trade rules that prioritize business interests and consequently harm public health.^189^ In particular, these companies lobbied trade representatives during negotiations for the Transpacific Partnership Agreement (TPP), an Asia‐Pacific trade agreement including Mexico, Peru, and Chile to further constrain policymakers' ability to propose and implement public health policies.^190^ More recently, food companies lobbied the United States to insert language during the North American Free Trade Agreement (NAFTA) renegotiations that would have caused significant difficulties in adopting FoP regulations,^191^ but because of public exposure and scrutiny, this provision was withdrawn.^192^

Food and beverage companies have also lobbied governments during Codex Alimentarius negotiations,^183^ which is a collection of international standards that establishes guidelines relating to food production and food safety. Although Codex is not a trade agreement, it is recognized by trade agreements and is referenced by governments during trade disputes in the World Trade Organization. Thus, transnational food and beverage companies attempt to alter Codex standards, which can in turn be used in trade disputes to globally preempt domestic public health policies.^184^ For example, they lobbied World Trade Organization member states to issue formal complaints against FoP policies in Chile, Ecuador, and Peru.^193^ Although these countries eventually moved forward with implementing FoP, policy implementation was delayed. Furthermore, other countries including Indonesia and Thailand were concerned with these trade disputes and weakened their policies.^193^

Although legal provisions exist in trade agreements to protect investors against government health regulations that are deemed unnecessary barriers to trade or violate a company's intellectual property rights, food and beverage companies purposely stretch these rules governing trade. Similar to transnational tobacco companies,^194^, ^195^ transnational food and beverage companies have threatened low‐ and middle‐income countries in Latin America in attempts to force them to withdraw public health proposals.^196^ While Uruguay is moving forward in implementing FoP, Costa Rican officials have announced they would prefer to avoid potentially costly legal battles.^196^

### 8.1 Solutions and strategies

Raising awareness about the economic, legal, and political impact of trade on food environments to the public, health advocates and policymakers can help governments better monitor trade developments and protect the policy space necessary to promote and protect healthy food environments where children thrive. Exposing trade activity of food and beverage companies in academic conferences, news media outlets, social media, public consultations, and trade negotiations can help minimize the advancement of business‐friendly trade rules that are harmful to public health. Additionally, public health departments should be involved when trade negotiations are conducted. The emerging literature that has systematically monitored how such practices and negotiations advance should invest in more clearly establishing the relationships between trade and health outcomes. For instance, a study that used country‐level sales data organized by Euromonitor International for the years of 2002 to 2016 showed that in countries that joined free trade agreements with the United States, sales of ultra‐processed foods increased by 0.89 kg per capita per annum and sales of baby foods increased by 0.17 kg per capita of children under age 5 per annum.^197^ Intersectoral governmental mechanisms that promote “health in all policies” can further help build the links between public health to other sectors of government, including, trade, finance, and agriculture.^198^

### 8.2 Research agenda

We identify three main areas of health‐related research: (i) analyze how existing trade developments impact food environments, diet, and, consequently, childhood obesity; (ii) examine new trade developments and rules that alter food environments; and (iii) assess how these agreements contribute to greater availability of the ultra‐processed foods mostly consumed by children and adolescents.^199^, ^200^ Given the role of large transnational food and beverage companies in shaping trade agreements, more evidence is needed on how they attempt to use existing trade agreements and influence new trade rules to negatively alter childhood obesity‐related food environments.

## 9 CONCLUSION

For Latin American children and Latino children living in the United States, the food environment is associated with food access, affordability, availability, and short‐ and long‐term food preferences and dietary quality. Using the INFORMAS framework, we have described ways in which different aspects of the food environment—including food retail, school food provision, food labeling and promotion, pricing, industry interference, and trade—promote excess intake of SSBs and other ultra‐processed foods among children, drivers of childhood obesity across regions. We argue that public policies are critical for improving the food environment of children living in Latin America and Latino children living in the United States. We present examples of policies that have been successfully implemented across the study regions, arguing for the need to expand them so more children benefit, in particularly those from low‐income settings. We also advocate for more rigorous evaluations of food policies considering equitable effects on children and potential unintended consequences. In Table A1, we provide a summary of the research priorities identified across the different food environments.

In addition, we identified critical gaps in knowledge cutting across all sectors that need to be addressed and guide the development of effective childhood obesity prevention policies. First, cross‐country comparisons—learning what works in one country and whether it could work well in another country—are missing. It is particularly relevant to understand the impact of food environment policies in Latin America because of the region's leadership in implementing innovative and comprehensive policies. Rigorous evaluations employing strong natural experimental designs and exploiting cross‐country differences in policy design can help inform which policies are most effective at shifting the food system and children's diets. Similarly, whether food environment policies that have worked in Latin America would be effective for Latino children in the United States remain unclear. Children and families from Latino heritage may respond differently given distinct commands of the English language and food environments (e.g., if SSB health warnings use English text, they may not be well understood by those with low English literacy).

The potential impact of food policies on obesity is somewhat modest when a single policy is applied alone,^201^, ^202^ which reinforces the need for sustaining action and combining regulatory measures to detect tangible progress in childhood obesity. In most cases, we assume that policies will have a unidirectional and single effect. However, in reality, childhood obesity is the result of multiple factors and actors that act interdependently, and therefore, using system science approaches that consider positive and negative feedback may help us understand real‐life impacts.^203^

In summary, we believe that a cross‐country, systems‐based approach is important from a research perspective so effective policies are better translated across borders, for framing expectations of policymakers, and rebutting messages of potential detractors of policies that can prevent childhood obesity, such as some actors from the food industry. Such an integrated body of evidence is still needed for the design and implementation of an optimal package of policies aimed at transforming the various aspects of a childhood obesity‐related food environment and the unequal access to healthy diets. Implementing these measures is particularly urgent and should be a priority of governments, academics, and the civil society to ensure children's right to a nutritious diet, as stated in the United Nations Convention on the Rights of the Child over 30 years ago.

## CONFLICT OF INTERESTS

No conflict of interest statement.

## APPENDIX A

**TABLE A1:** Research questions and methodological needs to advance the understanding of the role of different components of the food environment and childhood obesity in Latin America and among Latino children living in the United States

Food environment component	Research questions and methodological needs
Food retail	• How do we examine the impact of different food retailers while controlling for the selectivity of shopping at specific retailers and considering the endogeneity of the food environment (e.g., retailers' choice to locate in a particular area)? • What is the impact of different strategies to improve healthier food purchases *inside* food retailers? For example, “nudges” to alter choice architecture, such as product placement, banning ultra‐processed foods from checkout, changes on promotional strategies of ultra‐processed foods, and others. • Need for more innovative methods such as eye‐tracking technology, consumer panels, and experiments using virtual and actual “grocery store” labs. • Implications for cross‐borders research: Would strategies like healthy food financing, which have been implemented in the United States, work in Latin America? Conversely, are retailer‐based strategies, such as Mexico's ban on junk food to minors, feasible in the United States?
Food promotion	• What is the impact of personalized marketing, particularly in digital media, and integrated marketing strategies? • What is the broader impact of child‐directed food marketing on children's rights (e.g., privacy and healthy development)? • How well are existing statutory marketing policies being enforced and monitored? • What are the cross‐border implications of marketing policies implemented in one country on other countries without regulations? • How has industry responded to policies? What strategies does industry use to avoid or push back against new policies?
Provision of foods in schools	• How well are school food policies being implemented? What are the factors (country‐level, state‐level, district‐level, and school‐level) that drive implementation? • What are the impacts of school food policies on shaping the food system, including the local economy and family farmers' livelihood? • How and to what extent can food procurement for national school feeding policies be used to shape the food supply? • In countries without existing school policies, what is the food environment within schools and how is it changing, including availability and quality of foods and beverages, clean drinking water, and presence of food marketing? • What are the differential impacts of free universal and targeted school feeding programs on children's diet, consumption of the foods offered as part of school meals, and stigma and consequently children's mental health? • What is the impact of policies, including banning sales and marketing of sugar‐sweetened beverages (SSBs) and ultra‐processed foods, setting school food nutritional standards, requiring procurement from local farms, and requiring that high percentages of school foods be minimally processed, on children's outcomes, including dietary intake (both at school and total dietary intake), education (including attendance, graduation, and other metrics), and health? • What are the impacts of bans on sales of competing foods inside schools in the uptake of free school lunches?
Food labeling	• How do front‐of‐package labeling regulations interact with other food‐related policies, such as restrictions on child‐directed marketing, restrictions on nutrition claims, or policies influencing price, with regard to influencing both industry response and consumer behavior? • What is the role of schools in transmitting understanding and use of front‐of‐package labels? • With regard to real‐world evaluation studies, to what extent are changes in intake of nutrients of concern and ultra‐processed foods driven by industry changes (e.g., product reformulation) or shifts in consumer behavior, or both? • What are potential unanticipated consequences of front‐of‐package labeling regulations? For example, do “high in sugar” labels lead to reductions in sugar but increases in noncaloric sweetener? • To what extent can lessons learned in Latin America, where front‐of‐package labeling regulations have spread rapidly, be applied to Latino parents and children in the United States, particularly regarding their impact on diet and health? • While most countries in Latin America have implemented a warnings‐style label on nutrients of concern (e.g., salt, sugar, and saturated fat), primarily to reduce purchases and intake of ultra‐processed foods, what is the impact of voluntary positive labels on minimally processed foods? • What is the ideal nutritional profile to underpin a front‐of‐package labeling system? Relatedly, what is the optimal coverage of products in a food supply carrying the label to inform consumers and improve the healthfulness of purchases? • Do front‐of‐package labeling policies improve disparities in childhood diet and health, including by income level, education, and (in the United States) race–ethnicity?
Food prices	• What is the impact of taxes on overall dietary intake, not only taxed items, or their direct substitutes, but on total diets? • How sustainable are taxes? Do consumers eventually return to pretax levels of purchasing, and how much time does that take? • Do tax policies reduce or exacerbate health inequalities? What are the potential unintended consequences of SSBs and ultra‐processed food taxes, and what policies or strategies can be used to mitigate these? • What is the impact of taxes on US Latinos and how does this compare to the impact of taxes in Latin American countries? • What are the impact of other pricing strategies, like adjustments to subsidies to increase purchases and intake of healthy foods, such as fruits and vegetables?
